# Update on Wild Poliovirus Type 1 Outbreak — Southeastern Africa, 2021–2022

**DOI:** 10.15585/mmwr.mm7215a3

**Published:** 2023-04-14

**Authors:** Elizabeth Davlantes, Sharon A. Greene, Farrell A. Tobolowsky, Oladayo Biya, Eric Wiesen, Fikru Abebe, Mesfin B. Weldetsadik, Victor A. Eboh, Mike N. Chisema, Balbina da Conceição Mário, Florian Tinuga, Patricia Mupeta Bobo, Colline Koline Chigodo, Ghanashyam Sethy, Jan-Marcus Hellström, Abdou Moumouni Goundara, Marie-Eve Burny, Jonas C. Mwale, Jaume Jorba, Koketso S. Makua, Wayne Howard, Lerato Seakamela, Samuel Okiror, Andrea Thompson, Asma Ali, Dhoud Samba, Chukwuemeka Agbo, Lusamba Kabamba, Anthony Kazoka, Delayo Laurel Zomahoun, Fadinding Manneh, Khalid Abdelrahim, Chris Kamugisha, Abubakar Sadiq Umar

**Affiliations:** ^1^Global Immunization Division, Center for Global Health, CDC; ^2^Center for Vaccine Equity, The Task Force for Global Health, Decatur, Georgia; ^3^Malawi Ministry of Health; ^4^Mozambique Ministry of Health; ^5^Tanzania Ministry of Health; ^6^Zambia Ministry of Health; ^7^Zimbabwe Ministry of Health and Child Care; ^8^UNICEF, New York, New York; ^9^Division of Viral Diseases, National Center for Immunization and Respiratory Diseases, CDC; ^10^Polio Reference Laboratory, National Institute for Communicable Diseases, Johannesburg, South Africa; ^11^Polio Team, Bill & Melinda Gates Foundation, Seattle, Washington; ^12^McKing Consulting Corporation, Chamblee, Georgia; ^13^World Health Organization, Geneva, Switzerland.

Since the Global Polio Eradication Initiative (GPEI) began in 1988, the number of wild poliovirus (WPV) cases has declined by >99.99%. Five of the six World Health Organization (WHO) regions have been certified free of indigenous WPV, and WPV serotypes 2 and 3 have been declared eradicated globally (*1*). WPV type 1 (WPV1) remains endemic only in Afghanistan and Pakistan (*2*,*3*). Before the outbreak described in this report, WPV1 had not been detected in southeastern Africa since the 1990s, and on August 25, 2020, the WHO African Region was certified free of indigenous WPV (*4*). On February 16, 2022, WPV1 infection was confirmed in one child living in Malawi, with onset of paralysis on November 19, 2021. Genomic sequence analysis of the isolated poliovirus indicated that it originated in Pakistan (*5*). Cases were subsequently identified in Mozambique. This report summarizes progress in the outbreak response since the initial report (*5*). During November 2021–December 2022, nine children and adolescents with paralytic polio caused by WPV1 were identified in southeastern Africa: one in Malawi and eight in Mozambique. Malawi, Mozambique, and three neighboring countries at high risk for WPV1 importation (Tanzania, Zambia, and Zimbabwe) responded by increasing surveillance and organizing up to six rounds of national and subnational polio supplementary immunization activities (SIAs).* Although no cases of paralytic WPV1 infection have been reported in Malawi since November 2021 or in Mozambique since August 2022, undetected transmission might be ongoing because of poliovirus surveillance gaps and testing delays. Efforts to further enhance poliovirus surveillance sensitivity, improve SIA quality, and strengthen routine immunization are needed to ensure that WPV1 transmission has been interrupted within 12 months of the first case, thereby preserving the WHO African Region’s WPV-free status.

## Detection of WPV1

**FIGURE 1:**
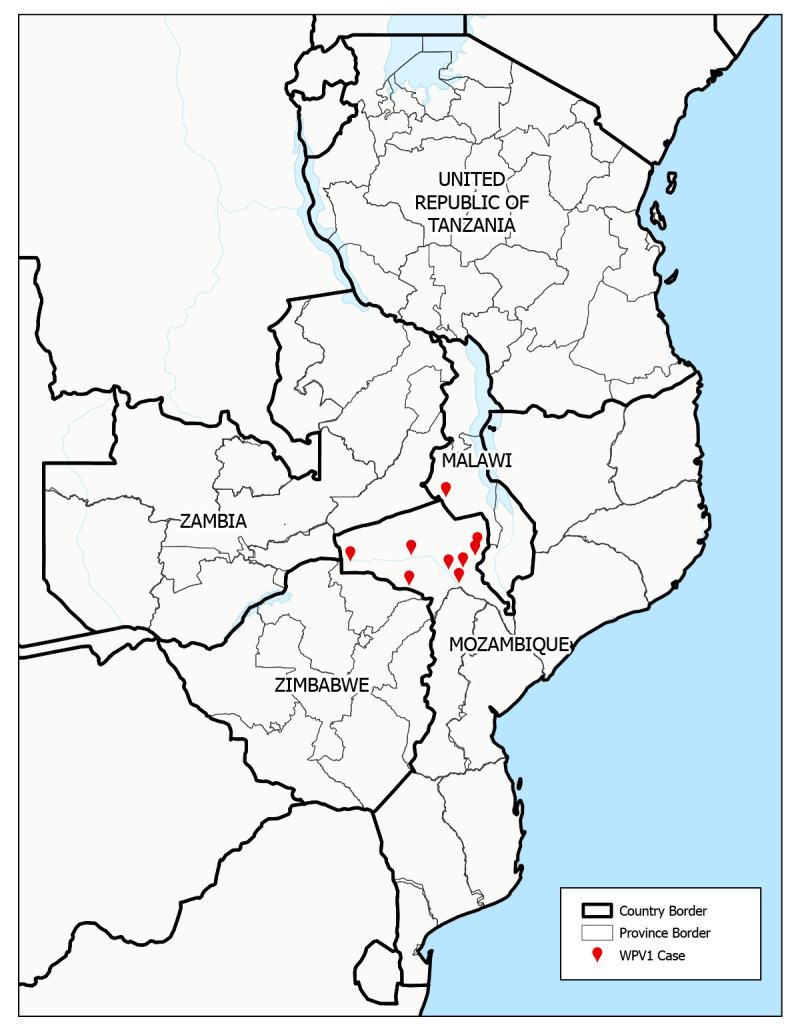
Location of wild poliovirus type 1 cases and the five outbreak response countries* — southeastern Africa, 2021–2022 **Abbreviation:** WPV1 = wild poliovirus type 1. * Malawi, Mozambique, Tanzania, Zambia, and Zimbabwe.

During November 2021–December 2022, and as of April 7, 2023, nine cases of paralytic polio caused by WPV1 had been detected in southeastern Africa. One case was previously reported in Lilongwe, Malawi (*5*), with onset of paralysis on November 19, 2021, and eight cases were identified in Mozambique (all in Tete Province in the country’s northwestern region) (Figure 1), with the latest onset on August 10, 2022. Several of the cases in Mozambique occurred in children and adolescents living close to international borders with Malawi, Zambia, and Zimbabwe. At paralysis onset, patient age ranged from 5 months to 14 years (median = 59 months). Five of the nine cases occurred in children and adolescents aged ≥5 years. Only two of the patients had received ≥3 doses of oral polio vaccine, the minimum required for adequate protection from type 1 polioviruses. Delays were reported in confirming all nine cases: a median of 53 days (range = 36–96 days) elapsed from the onset of paralysis to reporting genomic sequencing results, primarily because of delays in international stool specimen transport but also because of delays in case detection and in testing of stool samples and isolates upon their receipt in the laboratories.

Genomic sequence analysis of all polioviruses isolated in this outbreak demonstrated that the closest relative was a WPV1 lineage detected in Sindh, Pakistan in October 2019 (*5*). Extensive nucleotide changes detected among outbreak isolates indicated that WPV1 had been circulating in southeastern Africa for approximately 2 years before detection of the Malawi case.

## Routine Immunization

Routine immunization of all children with a polio vaccine is the cornerstone to prevention of disease transmission worldwide. In 2021, all five countries involved in this response had gaps in their routine immunization coverage for bivalent oral polio vaccine (bOPV, containing Sabin-strain serotypes 1 and 3), with none reaching the 90% national target recommended by WHO’s Global Vaccine Action Plan.^†^ In 2021, national coverage estimates for 3 doses of bOPV were 89% in Malawi, 67% in Mozambique, 70% in Tanzania, 87% in Zambia, and 86% in Zimbabwe. Efforts to strengthen polio routine immunization were made in all countries throughout 2022; the July 2023 release of 2022 global coverage estimates by WHO and UNICEF is anticipated to evaluate the success of these interventions.

## Poliovirus Surveillance

Poliovirus transmission is detected primarily through surveillance for acute flaccid paralysis (AFP) among children and adolescents aged <15 years accompanied by testing of stool specimens at a WHO-accredited laboratory in the Global Polio Laboratory Network. Two core AFP surveillance performance indicators are the nonpolio AFP (NPAFP) rate^§^ and stool adequacy rate^¶^ (*6*). During 2022, GPEI supported ministries of health in the two affected and three at-risk countries by deploying staff members to high-risk districts. There they assisted with conducting active surveillance visits to health facilities, sensitizing local clinicians and public health workers regarding AFP recognition and reporting, conducting community case searches for additional children and adolescents with paralysis, performing AFP case investigations and follow-up exams, fast-tracking sample transportation from the point of collection to the laboratory, and conducting other surveillance-strengthening activities.

**FIGURE 2:**
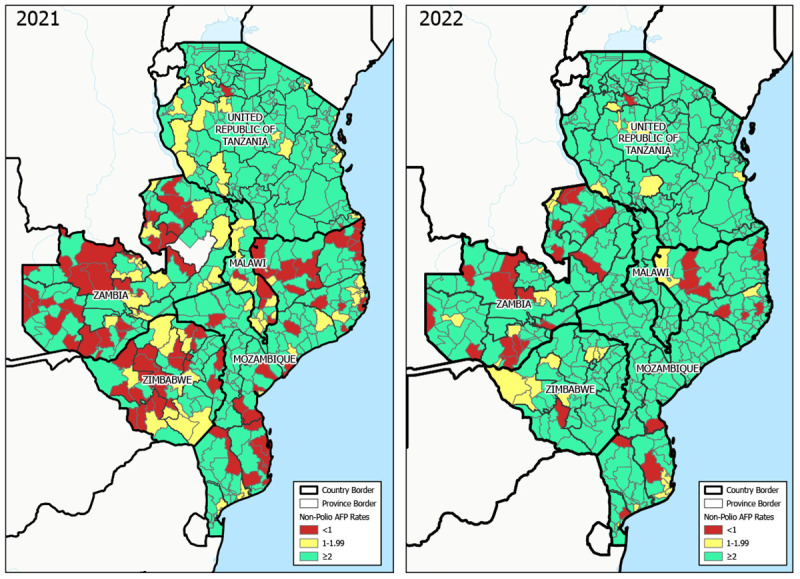
Nonpolio acute flaccid paralysis rates,* by district in the five outbreak response countries^†^ — southeastern Africa, 2021–2022 **Abbreviation**: AFP = acute flaccid paralysis. * Cases per 100,000 children and adolescents aged <15 years. ^†^ Malawi, Mozambique, Tanzania, Zambia, and Zimbabwe.

An NPAFP rate of two or more cases per 100,000 population aged <15 years is the global benchmark for surveillance that is sufficiently sensitive to detect a case of polio. During 2021 (before detection of the Malawi case), review of NPAFP surveillance performance in the outbreak-response countries identified many gaps at the district level (Figure 2). Among 554 districts within the five countries, 370 (67%) achieved this benchmark NPAFP rate. During 2022, subnational NPAFP rates improved markedly; 512 (92%) districts achieved the benchmark.

Among the five countries, two (Tanzania and Zimbabwe) achieved national-level stool adequacy rates of ≥80% in 2021 (98.5% and 91.5%, respectively) and 2022 (98.2% and 91.3%, respectively). However, national data can obscure considerable gaps in stool adequacy at the district level. For example, in 2022, 96% of districts in Tanzania and 87% in Zimbabwe achieved the 80% benchmark. In Malawi, Mozambique, and Zambia, this benchmark was achieved in only 43%, 52%, and 34% of districts, respectively. Without adequate stool specimens being delivered for laboratory analysis, additional cases of polio cannot be detected, and the full scope of the WPV1 outbreak cannot be known.

Environmental surveillance (testing of sewage for poliovirus) can supplement AFP surveillance. Environmental surveillance sites are considered reliably functioning when ≥50% of samples collected yield nonpolio enteroviruses (NPEVs). Before the outbreak, environmental sampling had been established in Mozambique, Tanzania, and Zambia; however, only Tanzania and Zambia had reliable, functioning environmental sampling programs. With intensive efforts by ministries of health and GPEI in 2022, environmental sampling was initiated in Malawi, and the number of sites in Mozambique, Tanzania, and Zambia was increased. The 50% NPEV detection target rate was achieved in six of 12 sites in Malawi, three of 14 in Mozambique, 10 of 11 in Tanzania, and 11 of 11 in Zambia. As of April 7, 2023, no sample collected at an environmental surveillance site during 2021–2022 in any of these countries has tested positive for WPV1.

## Supplementary Immunization Activities

With technical assistance from GPEI, the five outbreak response countries initiated a series of SIAs with bOPV within 33 days of the notification of WPV1 in Malawi; the SIAs targeted children aged <5 years. Four countries (Malawi, Mozambique, Tanzania, and Zambia) synchronized their initial response SIAs conducted in March 2022. However, subsequent rounds were not synchronized because of a variety of logistical constraints. In total, during 2022, Malawi conducted four national rounds, Tanzania and Zambia each conducted three, and Mozambique and Zimbabwe each conducted two. Subnational rounds were conducted in Mozambique (four), Tanzania (one), and Zambia (one).** During the sixth SIA in Mozambique, the target age range was increased to children and adolescents aged <15 years from children aged <5 years, because five of Mozambique’s eight WPV1 cases were detected in patients aged ≥5 years.

**FIGURE 3:**
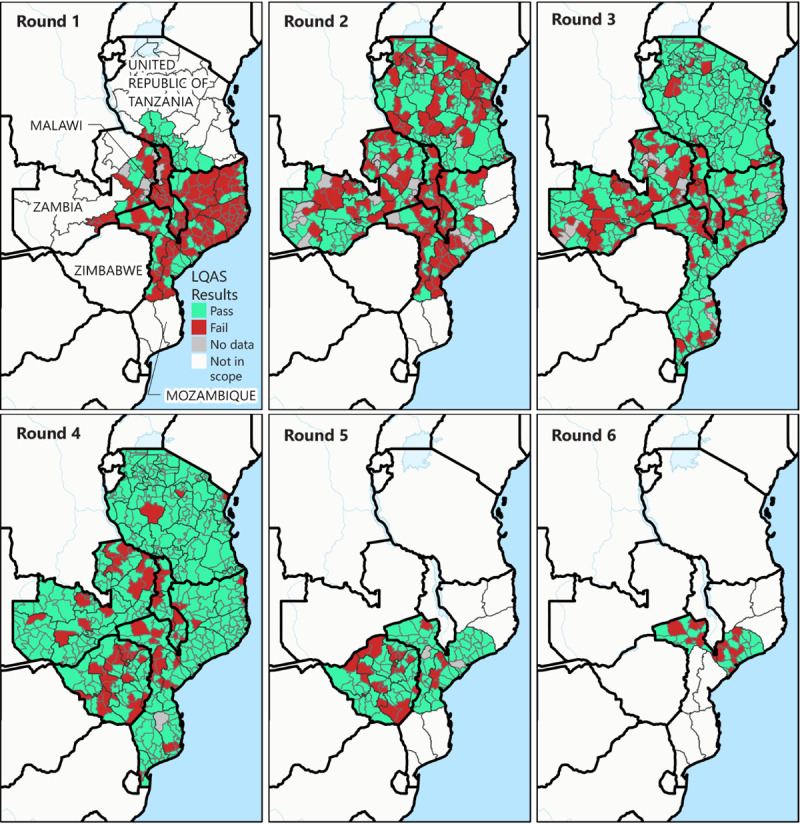
Bivalent oral poliovirus vaccine supplementary immunization activity quality as assessed by lot quality assurance sampling surveys, by supplementary immunization activity and district in the five outbreak response countries* —southeastern Africa, 2022 **Abbreviation**: LQAS = lot quality assurance sampling. * Malawi, Mozambique, Tanzania, Zambia, and Zimbabwe.

The quality of the SIAs was assessed by lot quality assurance sampling (LQAS) surveys within a week of SIA completion. In each district (lot) surveyed, six settlements were randomly selected, and 10 children from the SIA’s target age group were randomly selected within each.^††^ If at least 57 of the 60 selected persons had been vaccinated, the SIA was considered to be high-quality in that district (i.e., the district passed, with evidence that coverage is approaching 90%).^§§^ After the first SIAs in Malawi, Mozambique, and Zambia, fewer than 35% of districts passed based on LQAS results; however, assessed quality improved substantially during subsequent rounds (Figure 3). By the third SIA round in Mozambique and Tanzania, and the fourth round in Malawi and Zambia, more than 70% of districts passed based on LQAS results. Zimbabwe’s SIA quality also improved between the country’s first and second rounds.

Importantly, three of the six districts in Malawi and Mozambique where WPV1 cases were found failed LQAS in 50% of their countries’ SIA rounds; failures occurred in both early and later rounds. In Mozambique, this included the Moatize district, where four of the nine WPV1 cases had been detected during this outbreak.

## Discussion

All countries, including those that are polio-free, must be vigilant against importation of poliovirus and establishment of local transmission. The risk for importation and local transmission has been demonstrated by WPV1 detection in Malawi and transmission in Mozambique. GPEI partners and ministries of health in the five southeastern African countries have exhibited efficient collaboration in this outbreak response, extending from the international coordination level to the dedicated frontline health workers. Government engagement has been high in all five countries, despite many competing priorities. The establishment of national-level emergency operations centers for poliovirus responses has facilitated increased coordination and collaboration among GPEI partners. Surge GPEI international and national technical support has augmented existing personnel, particularly when addressing hard-to-reach areas and strengthening AFP surveillance efforts for timely detection.

Alongside these successes, the WPV1 outbreak response in southeastern Africa has faced several challenges. Although WPV1 transmission appears to have been interrupted, the identification of circulating vaccine-derived poliovirus (cVDPV) type 1 in Malawi and Mozambique during 2022 indicates ongoing local susceptibility to type 1 poliovirus (*7*). Global bOPV supply limitations led to delayed or smaller-scale SIAs in the outbreak response countries, which might not have been of sufficient magnitude to stop WPV1 transmission. Results of LQAS surveys in the six districts where WPV1 cases were reported indicate that SIAs might also have been of insufficiently high quality to stop transmission. In addition, cocirculation of cVDPV type 1 in Malawi and Mozambique and cVDPV type 2 in Mozambique and Zambia led to resource and logistical constraints in the response to the WPV1 outbreak (*7*). Prevention of further cross-border transmission could be accomplished through continued improved collaboration among bordering countries.

The findings in this report are subject to at least three limitations. First, gaps in poliovirus surveillance and delays in specimen testing could result in ongoing undetected transmission. Second, routine immunization coverage estimates are based on administrative data and might be inaccurate because of errors in recording doses administered or in estimating the target population; as a result, gaps in immunity might be underestimated. Finally, LQAS survey results might not be representative of the true proportion of children reached during each SIA.

The WHO Africa Regional Certification Commission has indicated that this outbreak does not currently affect Africa’s WPV-free certification, because it occurred after virus importation from Pakistan. However, if transmission continues ≥12 months after outbreak confirmation, this certification is at risk. For the WHO African Region to remain WPV-free, intensified efforts should focus on enhancing surveillance sensitivity and timeliness, improving SIA quality, and strengthening routine immunization efforts.

**Summary**

**What is already known about this topic?**

The World Health Organization (WHO) African Region was certified as having interrupted indigenous wild poliovirus (WPV) transmission in August 2020. In 2022, an outbreak of WPV type 1 (WPV1) was detected in southeastern Africa.

**What is added by this report?**

To date, one WPV1 case was detected in Malawi and eight in Mozambique. These countries and Tanzania, Zambia, and Zimbabwe implemented up to six national and subnational supplementary immunization activities (SIAs) per country and strengthened poliovirus surveillance.

**What are the implications for public health practice?**

Further enhancing surveillance, implementing high-quality SIAs, and strengthening routine immunization are essential to stopping WPV1 transmission within 12 months of the first case, thereby preserving the WHO African Region’s WPV-free status.

## Footnotes

* Polio SIAs are mass immunization campaigns intended to interrupt poliovirus circulation, usually by immunizing every child aged <5 years with 2 oral polio vaccine doses, regardless of previous immunization status.

†
https://www.who.int/publications/i/item/global-vaccine-action-plan-2011-2020

§ The NPAFP rate is an indicator of surveillance sensitivity; the global benchmark is detection of two or more NPAFP cases per 100,000 children and adolescents aged <15 years per year in nonoutbreak conditions, and three or more NPAFP cases per 100,000 children and adolescents aged <15 years per year during outbreaks.

¶ Stool adequacy refers to the percentage of AFP patient stool specimens that are collected within 14 days of paralysis onset and arrive at the laboratory in good condition. Stool adequacy rates ≥80%, the global benchmark, demonstrate that poliovirus can be effectively isolated from AFP stool samples if present.

** By country, national and subnational rounds occurred during the following approximate date ranges: Malawi (March 21–October 16); Mozambique (March 21–December 11); Tanzania (March 24–December 4); Zambia (March 21–October 30); and Zimbabwe (October 27–December 4).

†† Because the sixth SIA round in Mozambique targeted children and adolescents aged <15 years, the LQAS surveys for this round targeted children and adolescents in this age group.

§§
https://polioeradication.org/wp-content/uploads/2016/09/Assessing-Vaccination-Coverage-Levels-Using-Clustered-LQAS_Apr2012_EN.pdf
